# *Nhlrc2* is crucial during mouse gastrulation

**DOI:** 10.1002/dvg.23470

**Published:** 2022-03-08

**Authors:** Anniina E. Hiltunen, Reetta Vuolteenaho, Veli‐Pekka Ronkainen, Ilkka Miinalainen, Johanna Uusimaa, Siri Lehtonen, Reetta Hinttala

**Affiliations:** ^1^ Medical Research Center Oulu and PEDEGO Research Unit University of Oulu and Oulu University Hospital Oulu Finland; ^2^ Biocenter Oulu University of Oulu Oulu Finland; ^3^ Clinic for Children and Adolescents Pediatric Neurology Unit, Oulu University Hospital Oulu Finland; ^4^ Department of Obstetrics and Gynecology Oulu University Hospital Oulu Finland

**Keywords:** amniotic folding, ectoderm, embryonic stem cells, epiblast, gastrulation, *Nhlrc2*

## Abstract

The loss of NHL repeat containing 2 (*Nhlrc2*) leads to early embryonic lethality in mice, but the exact timing is currently unknown. In this study, we determined the time of lethality for *Nhlrc2* knockout (KO), C57BL/6NCrl‐*Nhlrc2* ^tm1a(KOMP)Wtsi^/Oulu, embryos and the *in situ* expression pattern of *Nhlrc2* based on *LacZ* reporter gene expression during this period. *Nhlrc2* KO preimplantation mouse embryos developed normally after *in vitro* fertilization. Embryonic stem (ES) cells established from KO blastocysts proliferated normally despite a complete loss of the NHLRC2 protein. *Nhlrc2* KO embryos from timed matings implanted and were indistinguishable from their wildtype littermates on embryonic day (E) 6.5. On E7.5, *Nhlrc2* KO embryo development was arrested, and on E8.5, only 6% of the genotyped embryos were homozygous for the *Nhlrc2* ^tm1a(KOMP)Wtsi^ allele. *Nhlrc2* KO E8.5 embryos showed limited embryonic or extraembryonic tissue differentiation and remained at the cylinder stage. *Nhlrc2* expression was ubiquitous but strongest in the epiblast/ectoderm and extraembryonic ectoderm on E6.5 and E7.5. NHLRC2 is essential for early postimplantation development, and its loss leads to failed gastrulation and amniotic folding in mice. Future studies on the evolutionarily conserved NHLRC2 will provide new insights into the molecular pathways involved in the early steps of postimplantation development.

## 1 INTRODUCTION

The NHL repeat containing 2 (*Nhlrc2*) gene encodes an evolutionarily conserved NHLRC2 protein consisting of a thioredoxin‐like, NHL‐repeat β‐propeller, and β‐stranded domains (Biterova, Ignatyev, Uusimaa, Hinttala, & Ruddock, 2018). Variants in this gene lead to a neurodegenerative and multiorgan disease called FINCA (OMIM #618278) (Brodsky et al., 2020; Rapp et al., 2021; Uusimaa et al., 2018). FINCA patients and mice harboring a similar genotype develop normally *in utero* and are healthy at birth, despite having only a low amount of affected NHLRC2 (Hiltunen et al., 2020; Uusimaa et al., 2018). However, several studies have implied an important, but still unknown, role of NHLRC2 in embryonic development across species. In bovine, p.Val311Ala substitution in the β‐propeller domain of NHLRC2 has been reported to cause neural tube‐related developmental malformations in Angus cattle (Denholm, 2017), and bovine oocytes with greater developmental competence have been reported to have higher *Nhlrc2* expression (Nemcova et al., 2016). *Nhlrc2* has also been associated with embryo–maternal crosstalk in ewes and bovine (Xiao et al., 2021; Q. Yang et al., 2020). The complete loss of *Nhlrc2* leads to early embryonic lethality in mice (Delhotal, 2016; Perez‐Garcia et al., 2018; Uusimaa et al., 2018), indicating its essential role in embryonic development. Only very few remnants of *Nhlrc2* KO trophoblast giant cells have been detected on embryonic day (E) 9.5 (Perez‐Garcia et al., 2018) but the exact time of lethality of the embryo is unknown.

The molecular function of NHLRC2 is still relatively poorly understood, but it has been connected with a variety of molecular pathways, including reactive oxygen species‐induced apoptosis in colon cancer cells (Nishi et al., 2017); vesicle transport, cytoskeleton organization, and endothelial to mesenchymal transition in fibroblasts (Paakkola et al., 2018); phagocytosis (Haney et al., 2018; Yeung et al., 2019) and actin dynamics (Haney et al., 2018) in human macrophages; and RNA metabolism in mouse neurons and hippocampus (Hiltunen et al., 2020). All these pathways are relevant to embryonic development, and a more detailed understanding of the early developmental defects arising from the loss of NHLRC2 can reveal new information on the early embryo development and function of NHLRC2.

In the current study, we determined the precise time of lethality for embryos lacking NHLRC2 to the time of gastrulation and describe the *in situ* expression pattern of *Nhlrc2* on E6.5 and E7.5. We also found that NHLRC2 is not essential for embryonic stem (ES) cells *in vitro*. The data presented here provide new information on the essential role of NHLRC2 during early postimplantation period of embryonic development.

## 2 RESULTS

### 2.1 The preimplantation development of *Nhlrc2* KO embryos is unaffected

**FIGURE 1:**
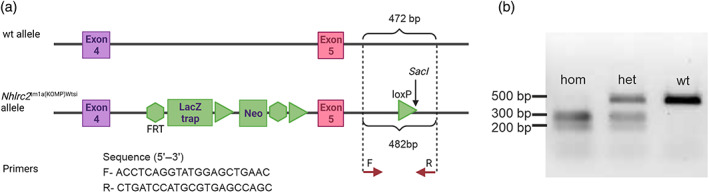
Genotyping scheme for *Nhlrc2* ^tm1a(KOMP)Wtsi^ allele. (a) The *SacI* restriction site in the synthetic loxP site is indicated. The PCR amplifies a 472 base pair (bp) region in the wt and a 482 bp region in the *Nhlrc2* ^tm1a(KOMP)Wtsi^ allele. The difference in length is due to the insertion of an 80 bp loxP site and a 70 bp deletion resulting from this insertion. Created with BioRender.com. (b) Gel electrophoresis of PCR products obtained from different genotypes after *SacI* digestion. The digestion leads to 197 bp and 285 bp fragments in the PCR product from *Nhlrc2* ^tm1a(KOMP)Wtsi^ allele. het,  heterozygous; hom,  homozygous

As part of our previous publication (Uusimaa et al., 2018), we observed that morula‐stage (E2.5) homozygous C57BL/6NCrl‐*Nhlrc2* ^tm1a(KOMP)Wtsi^/Oulu embryos (*Nhlrc2* KO hereafter) (originally obtained from European Mouse Mutant Archive EMMA ID: EM:10219) (Skarnes et al., 2011; The International Mouse Knockout Consortium, 2007) were absent after heterozygous breeding. Our recent studies have confirmed that the *Nhlrc2* ^tm1a(KOMP)Wtsi^ allele indeed leads to complete loss of full‐length *Nhlrc2* without any leakage (Hiltunen et al., 2020) and can be used as an *Nhlrc2* KO allele to study its effect during embryogenesis. We performed *in vitro* fertilization (IVF) with heterozygous gametes and out of the 96 embryos obtained, 82 (85.4%) developed to the blastocyst stage and attached to gelatinized plates, forming a colony in the following 10‐day culture *in vitro*. This proportion was close to the 86.4% of embryos that formed a colony in the control IVF using wildtype (wt) C57BL/6NCrl gametes. Next, we designed the genotyping protocol to enable identification of all three genotypes from one polymerase chain reaction (PCR) product utilizing a *SacI* digestion site present in the targeting construct (Figure 1). The genotype was determined for 60 IVF‐derived embryos, which, revealed a normal Mendelian ratio of *Nhlrc2* KO embryos (Table 1). Previously, the morulae were obtained from *in vivo* fertilization (IVO) on E2.5, and we decided to re‐genotype 15 of them with the new genotyping protocol to confirm that the IVF did not promote the development of the *Nhlrc2* KO embryos. The genotyping revealed that 20% of the IVO embryos were homozygous *Nhlrc2* KO embryos (Table 1), contrary to our previous report. This was further verified by Sanger sequencing.

**TABLE 1:** Genotyping summary of embryos obtained from heterozygous *Nhlrc2* KO mouse IVF and IVO matings

	WT % (*N*)	HET % (*N*)	HOM % (*N*)
Expected	25	50	25
IVF (*N* = 60)	20 (12)	45 (27)	35 (21)
IVO (*N* = 15)	20 (3)	60 (9)	20 (3)
E6.5 (*N* = 34)	20.6 (7)	52.9 (18)	26,5 (9)
E7.5 (*N* = 36)	27.8 (10)	47.2 (17)	25 (9)
E8.5 (*N* = 34)^a^	35.3 (12)	58.8 (20)	5.9 (2)

HET,  heterozygous; HOM,  homozygous; IVF,  in vitro fertilization; IVO,  in vivo fertilization; WT,  wildtype.

^a^ Deviates from Mendelian ratio (*p* = .031, *χ* ^2^ test).

These results indicate that, against our previous data, preimplantation development is not compromised by the loss of *Nhlrc2* in mice.

### 2.2 Mouse ES cells are viable without *Nhlrc2*

**FIGURE 2:**
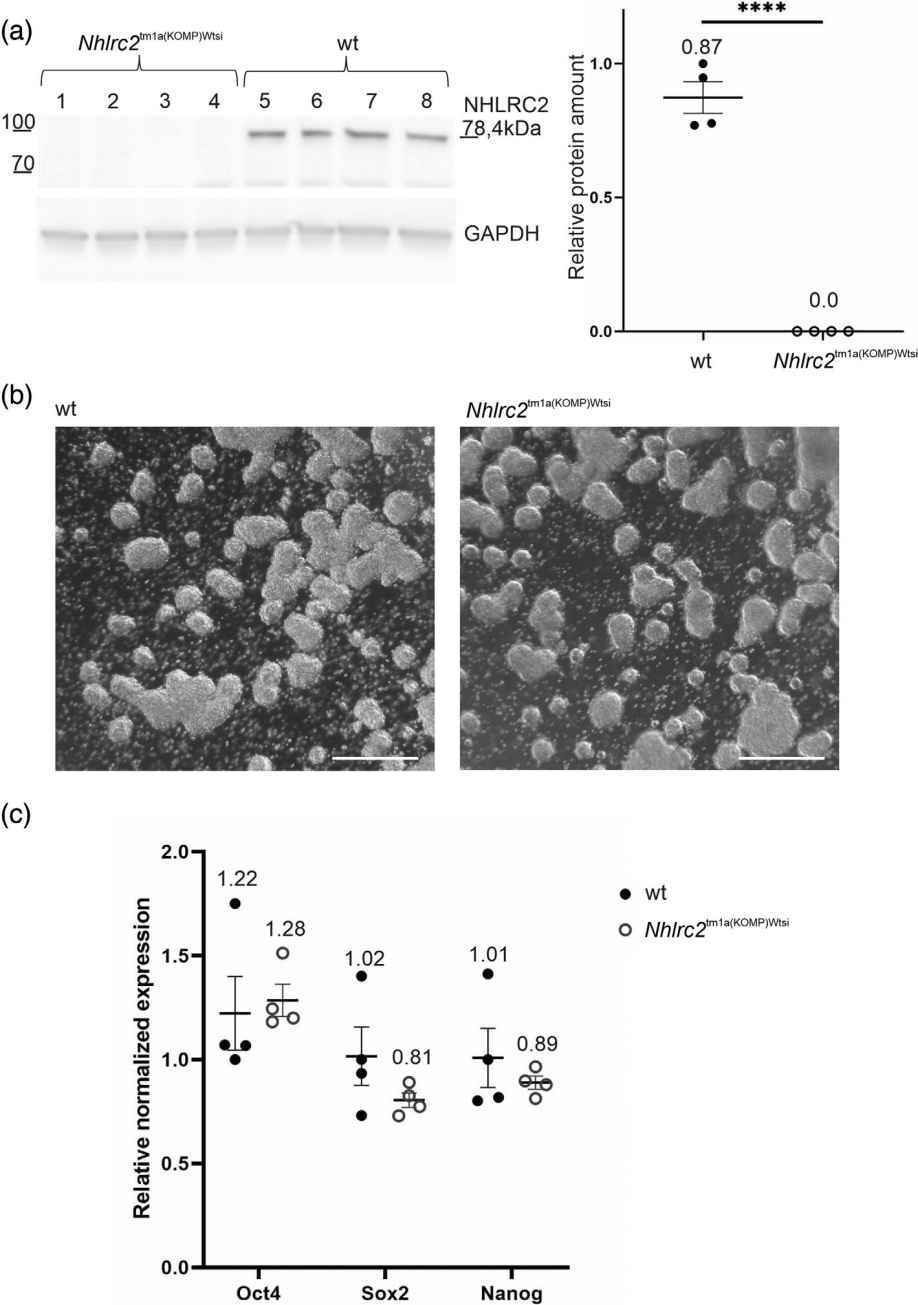
Mouse ES cells survive without NHLRC2. (a) Immunoblotting of four homozygous *Nhlrc2* ^tm1a(KOMP)Wtsi^ and four wt ES cell lines established from separate embryos. No NHLRC2 protein was detected in homozygous *Nhlrc2* ^tm1a(KOMP)Wtsi^ ES cells. Protein amounts are relative to one of the wt samples, and GAPDH was used for normalization. (b) Light microscope image of *Nhlrc2* ^tm1a(KOMP)Wtsi^ and wt ES cells cultured in suspension at the time of RNA isolation. Scale bar: 500 μm. (c) The expression levels of *Oct4*, *Sox2*, and *Nanog* in four *Nhlrc2* ^tm1a(KOMP)Wtsi^ and four wt ES cell lines established from separate embryos showed no statistically significant differences. Expression is relative to one of the wt samples, and *Gapdh*, and *Actb* were used as reference genes. Statistical analyses were conducted using Student's *t* test, ****p < .0001. Scatter plots show the individual data points, group means, and standard error of means (*SEM*)

As *Nhlrc2* KO preimplantation embryos formed normal colonies *in vitro*, we next established four wt and four homozygous *Nhlrc2* ^tm1a(KOMP)Wtsi^ ES cell lines from separate IVF‐derived embryos using the 2i method (Nichols, Silva, Roode, & Smith, 2009). The *Nhlrc2* ^tm1a(KOMP)Wtsi^ ES cells showed no NHLRC2 in immunoblotting, confirming the complete loss of protein in the *Nhlrc2* ^tm1a(KOMP)Wtsi^ clones (Figure 2a). The *Nhlrc2* ^tm1a(KOMP)Wtsi^ ES cells appeared normal (Figure 2b) and had similar expression levels of pluripotency markers *Oct4*, *Sox2*, and *Nanog* (Figure 2c) compared with wt cells. This indicates that NHLRC2 is not essential for mouse ES cells.

### 2.3 Development of *Nhlrc2* KO embryos fails after E6.5

**FIGURE 3:**
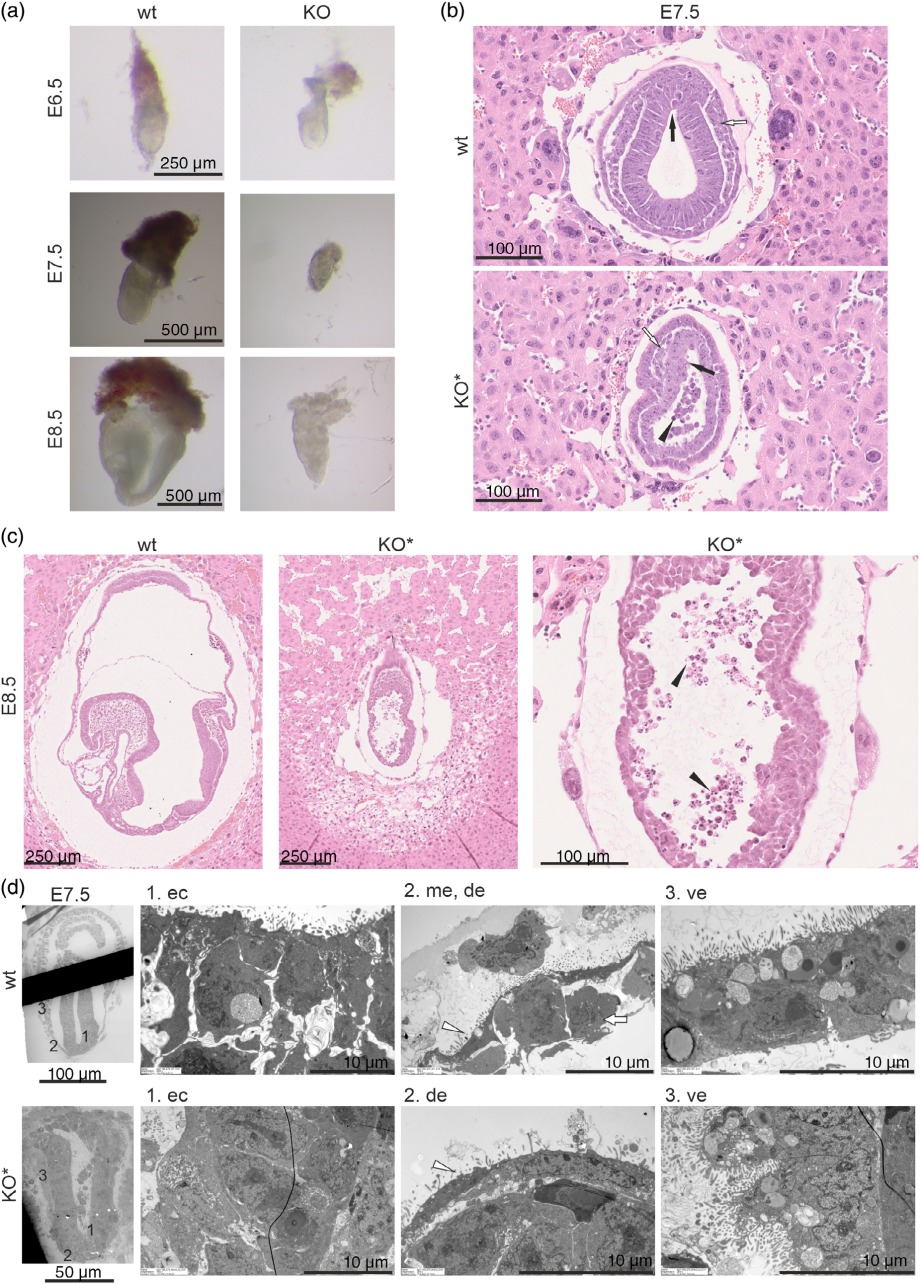
*Nhlrc2* KO embryos fail to develop after E6.5. (a) *Nhlrc2* KO and wt littermate on E6.5, E7.5, and E8.5. (b) Cross‐section of presumed *Nhlrc2* KO and wt embryo on E7.5 stained with hematoxylin and eosin. Primitive streak (black arrow), forming mesoderm (white arrow), and apoptotic cells (black arrowhead) are indicated. KO* embryos are presumed to be *Nhlrc2* KO based on their phenotypic features that are similar to homozygote *Nhlrc2* KOs, which have been genotyped. (c) Sagittal section of a normal E8.5 embryo and a presumed *Nhlrc2* KO embryo on E8.5 stained with hematoxylin and eosin. Higher magnification of the presumed *Nhlrc2* KO embryo showing apoptotic cells (black arrow). KO* embryos are presumed to be *Nhlrc2* KO based on their phenotypic features. (d) TEM image of a wt and presumed *Nhlrc2* KO E7.5 embryo. Higher magnification of ectoderm (ec), mesoderm (me) (white arrow), definitive endoderm (de) (white arrowhead), and visceral endoderm (ve) cells are shown. KO* embryos are presumed to be *Nhlrc2* KO due to their phenotype and have not been genotyped

As shown, the preimplantation development of homozygous *Nhlrc2* KO embryos is not affected, and a previous report of this specific mouse line has shown that the KO embryos are no longer present on E9.5 (Perez‐Garcia et al., 2018). To determine the exact time of lethality between implantation and E9.5, we isolated and genotyped early postimplantation embryos starting from E6.5 from the timed matings of heterozygous *Nhlrc2* KO mice. Out of 34 isolated embryos, seven were *Nhlrc2* KO (Table 1). The *Nhlrc2* KO embryos did not show any apparent defects with comparable size and ectoplacental cone appearance with their wt littermates, indicating normal implantation (Figure 3a). On E7.5, the homozygous *Nhlrc2* KO embryos were still present in the expected ratio according to Mendelian inheritance, but they were developmentally arrested or even degenerated (Figure 3a and Table 1). The developing amnion and chorion were not visible in the *Nhlrc2* KO embryos through stereomicroscope examination. On E8.5, we obtained only two homozygous *Nhlrc2* KO embryos out of the 34 isolated embryos (5.9%) (Table 1). In addition, four deciduae with only small remnants of an embryo were collected but could not be reliably genotyped. The development of the two confirmed *Nhlrc2* KO embryos was severely arrested, and their gross morphology resembled that of an E6.5 rather than an E8.5 embryo (Figure 3a). Although the *Nhlrc2* KO embryos grew in size to approximately that of an E7.5 embryo, no defined internal structures could be detected.

The genotyped *Nhlrc2* KO embryos were distinguishable from their wt littermates from E7.5 onwards, based on their poor appearance. We then selected similar poorly surviving, presumably *Nhlrc2* KO embryos, for further structural analysis with their wt littermates. We examined the ultrastructure of E7.5 embryos using transmission electron microscopy (TEM) and the morphology of E7.5 and E.8.5 embryos from hematoxylin eosin‐stained histological sections. The TEM images on E7.5 did not show any abnormalities between the poorly surviving and normal embryos in the intracellular ultrastructure of the ectoderm, definitive endoderm, or visceral endoderm (Figure 3d). Moreover, organized mesoderm or ectoderm layers were not detected in the presumed *Nhlrc2* KO embryos. Cross‐sections of E7.5 embryos revealed loss of normal columnar morphology of the ectoderm and the appearance of apoptotic cells within the proamniotic cavity in the presumed *Nhlrc2* KO embryos. Although the embryos were delayed compared to wt embryos, they had developed cells suggestive of primitive streak formation and mesoderm initiation (Figure 3b). On E8.5, the presumed *Nhlrc2* KO embryos remained at the egg cylinder stage and again lacked organized mesoderm and ectoderm layers, allantois, amnion, chorion, and embryonic structures, such as headfold, and showed massive apoptosis (Figure 3c).

In sum, our results show that loss of NHLRC2 leads to the termination of embryonic development after E6.5 in mice at the time of gastrulation.

### 2.4 *Nhlrc2* ^tm1a(KOMP)Wtsi^ ES cells show normal expression of differentiation markers after spontaneous differentiation

**FIGURE 4:**
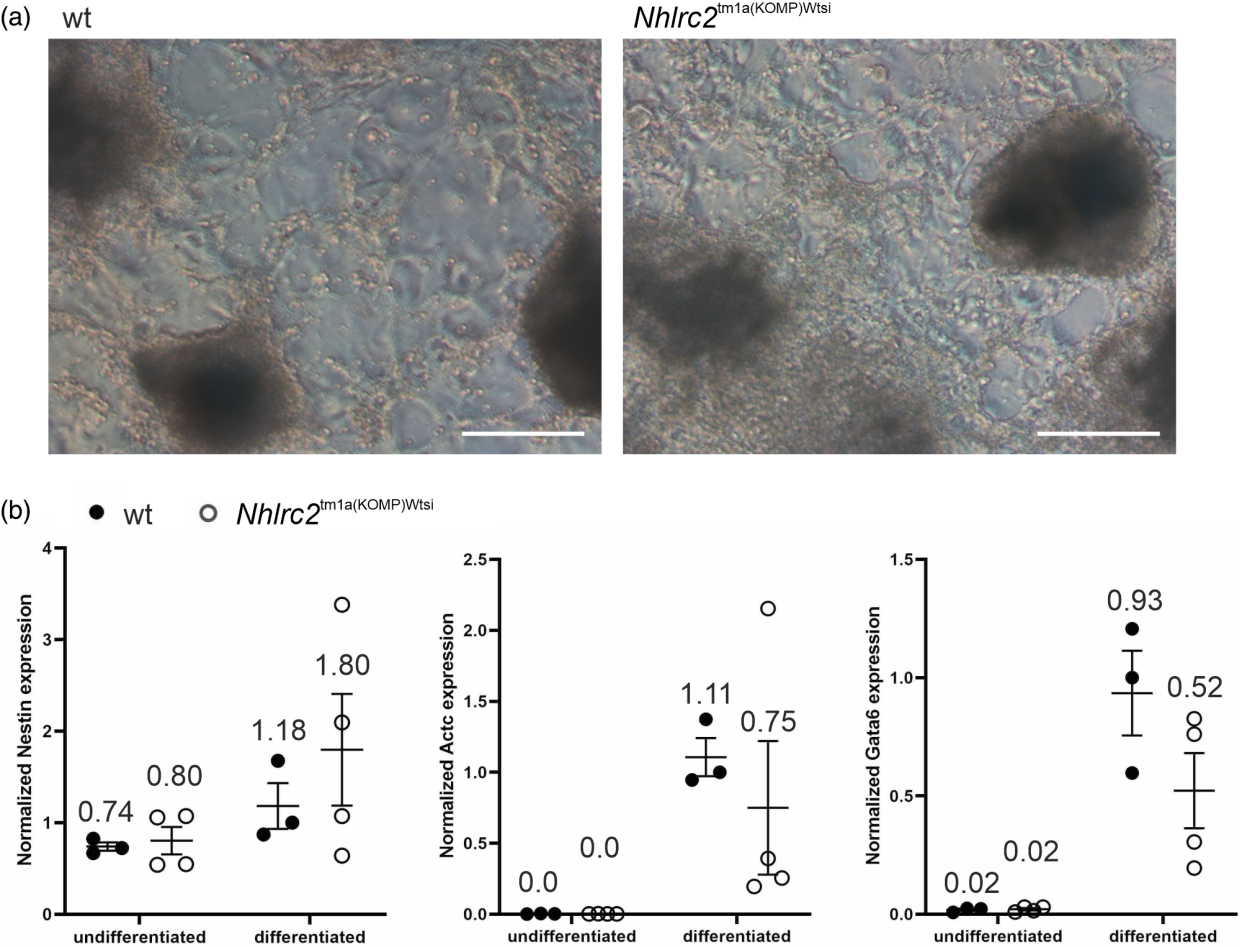
Spontaneous differentiation of *Nhlrc2* ^tm1a(KOMP)Wtsi^ and wt ES cells. (a) Image of *Nhlrc2* ^tm1a(KOMP)Wtsi^ and wt ES cells after 16 days of spontaneous differentiation. Scale bar: 250 μm. (b) Expression levels of *Nestin*, *Actc*, and *Gata6* in four *Nhlrc2* ^tm1a(KOMP)Wtsi^ and three wt ES cell lines established from separate embryos. Expression is relative to one of the differentiated wt samples, and *Gapdh* and *Pkg1* were used as reference genes. Statistical analysis was conducted using Student's *t* test and showed no statistically significant differences. Scatter plots show the individual data points, group means, and *SEM*

As the *Nhlrc2* KO embryos failed at the time of gastrulation and did not show a normal ectoderm and mesoderm organization, we cultured *Nhlrc2* ^tm1a(KOMP)Wtsi^ and wt ES cells in spontaneous differentiation conditions to determine whether the *Nhlrc2* ^tm1a(KOMP)Wtsi^ cells had the potential to differentiate toward all three germ layers. According to quantitative polymerase chain reaction (qPCR) analysis of four *Nhlrc2* ^tm1a(KOMP)Wtsi^ and three wt ES cell clones for the ectoderm (*Nestin*), mesoderm (*Actc*), and endoderm (*Gata6*) markers after 16 days of differentiation, the *Nhlrc2* ^tm1a(KOMP)Wtsi^ cells expressed these markers comparable to their wt counterparts (Figure 4). Thus, although a normal cell layer organization was not detected in the presumed *Nhlrc2* KO embryos *in vivo*, the *Nhlrc2* ^tm1a(KOMP)Wtsi^ ES cells were able to initiate the differentiation toward all germ layer lineages *in vitro*.

### 2.5 *Nhlrc2 in situ* expression on E6.5 and E7.5

**FIGURE 5:**
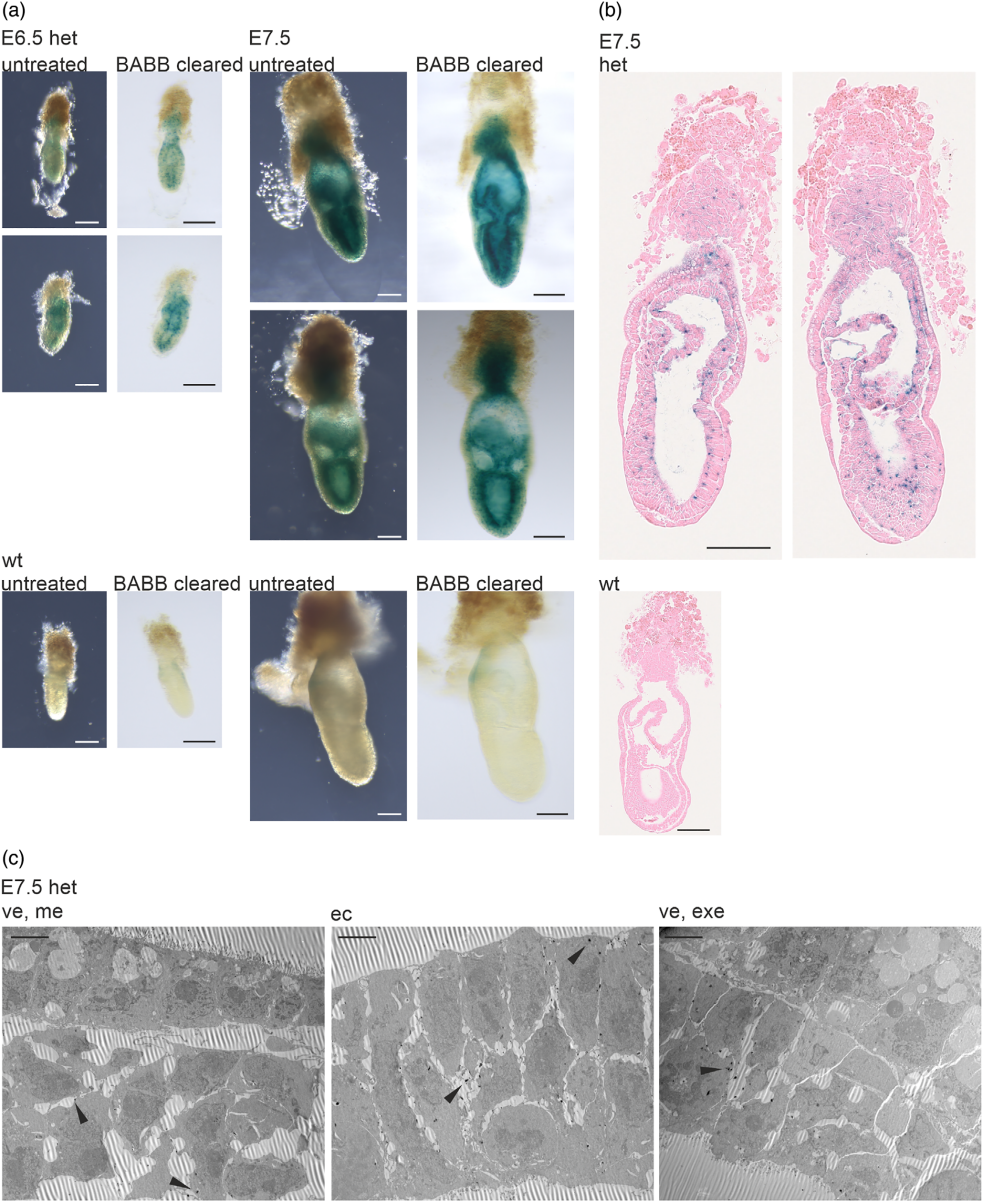
*Nhlrc2* expression on E6.5 and E7.5 in mouse embryo. (a) β‐galactosidase‐stained heterozygous *Nhlrc2* KO and wt embryos on E6.5 and E7.5. Staining is strongest in the epiblast and ectoderm layers. The same embryo imaged before (untreated) and after BABB clearing. Scale bar: 100 μm. (b) Sections of β‐galactosidase‐stained heterozygous and wt embryos on E7.5 counterstained with eosin. Two different planes of the same heterozygous *Nhlrc2* KO embryo are shown. Staining was strongest in the ectoderm. Mesoderm and ectoplacental cones were moderately stained. A small amount of endodermal staining was present, mostly adjacent to the ectoplacental cone. Scale bar: 100 μm. (c) TEM images of β‐galactosidase‐stained heterozygous E7.5 mouse embryo. Arrows indicate the X‐gal reaction product most often seen in the ectoderm cells, moderately seen in the mesoderm cells, and almost not found in the endoderm cells. Scale bar: 5 μm. ve, visceral endoderm; me, mesoderm; ec, ectoderm; exe, extraembryonic ectoderm

The examined *Nhlrc2* KO mouse line contains an IRES:*lacZ* trapping cassette that can be used as a reporter for *Nhlrc2* expression (Figure 1a), thus compensating for the current lack of specific antibody against mouse NHLRC2 in immunohistochemistry. We performed whole‐mount β‐galactosidase staining of heterozygous *Nhlrc2* KO embryos on E6.5 and E7.5 to determine the expression pattern of *Nhlrc2 in situ*. The heterozygous mice are viable and show no apparent phenotype (Uusimaa et al., 2018). The staining revealed a widespread *Nhlrc2* expression at both time points (Figure 5a), consistent with previous single‐cell RNA sequencing data from gastrulation and the early stages of the organogenesis of mice (Pijuan‐Sala et al., 2019). The strongest staining was observed in the epiblast/ectoderm and extraembryonic ectoderm on E6.5 and E7.5 (Figure 5a). On E7.5, the mesoderm and ectoplacental cone showed moderate staining, whereas the definitive and visceral endoderm were almost unstained (Figure 5b). This was also confirmed by TEM, in which the X‐gal reaction product could be detected in larger quantities in both embryonic and extraembryonic ectoderm cells compared with mesoderm cells, and almost no reaction product was present in the visceral and definitive endoderm on E7.5 (Figure 5c). Developing amnion and allantois were also strongly stained on E7.5 (Figure 5a,b).

## 3 DISCUSSION

Our results show that loss of NHLRC2 leads to the termination of embryonic development after E6.5 in mice and its loss is detrimental to amniotic folding and the completion of gastrulation. We also show that *Nhlrc2* is widely expressed in the gastrulating embryo, with the strongest expression in the embryonic and extraembryonic ectoderms. However, *Nhlrc2* is not essential for mouse ES cells and their spontaneous differentiation.

The halt in development happened considerably later than we expected in the basis of our previous results (Uusimaa et al., 2018). Previous attempts to establish cell lines with complete loss of *Nhlrc2* in THP‐1 cells (Yeung et al., 2019) and human fibroblasts (Paakkola et al., 2018) have yielded only knockdown cells, highlighting the essentiality of NHLRC2 in differentiated cells. Here, the establishment of *Nhlrc2* ^tm1a(KOMP)Wtsi^ ES cell clones provided an important access to *Nhlrc2* null control and enabled further optimization of the genotyping protocol. The new improved protocol, described in the current study, identifies all three genotypes from one PCR amplified product and thus requires less DNA template. This is crucial when genotyping such small samples.

The *Nhlrc2* KO mouse embryos showed no normal ectoderm and mesoderm cell layer organization or amniotic folding. This could be an indication of a defective Nodal, bone morphogenic protein, transforming growth factor beta (TGF‐β), or Wnt signaling (Tam & Loebel, 2007), as impaired mesoderm induction can result in loss of internal embryonic or extraembryonic structure development. For example, embryos lacking *Smad2* or *Smad4*, a TGF‐β mediator, have defective mesoderm induction and show some similarity to the phenotype described here (Sirard et al., 1998; Weinstein et al., 1998; X. Yang, Li, Xu, & Deng, 1998). However, in contrast to *Nhlrc2* KO embryos, these mutants also showed a defective egg cylinder formation not evident in *Nhlrc2* KO embryos. The thioredoxin‐like domain, also found in NHLRC2, is characteristic to oxidoreductases such as thioredoxin. Conversely, thioredoxin KO mice die shortly after implantation before E6.5, and their inner cell masses fail to proliferate *in vitro* (Matsui et al., 1996).

The observation that NHLRC2 is not essential for mouse ES cells, raises the question of whether NHLRC2 may be critical for cell‐to‐cell communication or cell migration during embryogenesis, which are not important in a monolayer culture. Decrease in NHLRC2 has been found to affect cytoskeleton organization and endocytic vesicle trafficking pathway *in vitro* (Haney et al., 2018; Paakkola et al., 2018; Yeung et al., 2019). Actomyosin and microtubule cytoskeletal systems are essential for the various cell behaviors needed for gastrulation movements (Solnica‐Krezel & Sepich, 2012). Endocytic pathway is required for signal amplification and termination for spatiotemporal regulation of cell movements during gastrulation (Wada & Sun‐Wada, 2013). Moreover, mutations leading to decreased cell proliferation can result in failed mesoderm induction and developmental arrest on E6.5 (Power & Tam, 1993; Tam & Behringer, 1997). For example, the KO of *Hnrnpc*, a gene whose expression we have previously found to be affected in the neuronal precursor cells of the NHLRC2 deficient FINCA mouse model (Hiltunen et al., 2020), also leads to an inability to develop past the cylinder stage but does not affect the viability of ES cells (Williamson, Banik‐Maiti, DeGregori, & Ruley, 2000).

For future studies, the strong ectodermal expression of *Nhlrc2* observed here may be of interest in relation to the neural tube defects seen in Angus cattle with NHLRC2 variants, considering that the ectoderm later forms neural tissues. Also, as the complete loss of *Nhlrc2* has proven difficult to attain in several cell types (Paakkola et al., 2018; Yeung et al., 2019), the *Nhlrc2* ^tm1a(KOMP)Wtsi^ ES cells enable a defined differentiation and further studies on the functional role of NHLRC2.

In conclusion, our findings highlight the importance of *Nhlrc2* in normal gastrulation. Further studies on this gene will determine the molecular pathways affected by NHLRC2 to reveal its potential role in early post implantation development and infertility.

## 4 METHODS

### 4.1 Animals

C57BL/6NCrl‐*Nhlrc2* ^tm1a(KOMP)Wtsi^/Oulu mice (EMMA ID: EM:10219) (Skarnes et al., 2011) were bred in a specific pathogen‐free facility, and all experiments were carried out in the conventional unit of the Oulu Laboratory Animal Center under conditions detailed previously (Hiltunen et al., 2020). All animal experiments were approved by the Regional State Administrative Agency of Southern Finland. All animal care and experimental procedures were conducted in adherence to European Union regulations and guidelines, Finnish legislation for housing laboratory animals (DIRECTIVE 2010/63/EU, Act 497/2013 and Decree 564/2013), and AAA's Guiding Principles in the Care and Use of Animals.

### 4.2 *In vitro* fertilization

IVF and embryo culture for 10 days on a gelatinized dish in an ES cell culture medium were performed at the Biocenter Oulu Transgenic and Phenotyping Core Facility (Oulu, Finland), as described previously (Hiltunen et al., 2020; Uusimaa et al., 2018).

### 4.3 DNA extraction and PCR

After trypsinization, the ES cell pellets were lysed with 0.1 mg/ml proteinase K in lysis buffer (0.1 M Tris pH 8.5, 5 mM ethylenediaminetetraacetic acid, 0.2% sodium dodecyl sulfate [SDS], 0.2 M NaCl), and the DNA was isolated through ethanol precipitation. E6.5–E8.5 DNA was isolated using 40 μl of QuickExtract (Lucigen, WI) solution according to the manufacturer's instructions, and 1–5 μL of the lysate was used for PCR.

PCR was used to amplify a region in *Nhlrc2*, where the insertion of the targeting construct resulted in a new *SacI* digestion site, with the primers shown in Table 2. Phire Hot Start II Polymerase (Thermo Fisher Scientific, Waltham, MA) and a Piko Thermal Cycler (Thermo Fisher Scientific, Vantaa, Finland) were used according to the manufacturer's instructions. Sanger sequencing was performed to validate the PCR product as described previously (Hiltunen et al., 2020). The PCR product from E6.5–8.5 embryos was precipitated with NaCl (4 M, 1:10) and ethanol before digestion overnight at −20°C or for 30 min at −70°C. The PCR product was digested using the *SacI* restriction enzyme (Thermo Scientific, Vilnius, Lithuania) according to the manufacturer's instructions and run on 1.5% agarose gel (BioNordika, Helsinki, Finland). SYBR Safe DNA Gel Stain (Invitrogen, Carlsbad, CA) was used for detection.

**TABLE 2:** Genotyping primers

Primer	Sequence (5′–3′)
Nhlrc2_F	ACCTCAGGTATGGAGCTGAAC
Nhlrc2_R	CTGATCCATGCGTGAGCCAGC

### 4.4 Establishment of mouse ES cell culture with the 2i method

Using the 2i method, ES cell cultures were established at the Biocenter Oulu Transgenic and Tissue Phenotyping Core Facility (Oulu, Finland) (Nichols et al., 2009). Embryos were isolated on E2.5 from super‐ovulated female oviducts after heterozygous *Nhlrc2* KO matings. The embryos were cultured overnight in KSOM (Merck, Darmstadt, Germany) supplemented with glycogen synthase kinase‐3 (CHIR 99021, Axon Medchem, Groningen, the Netherlands) and MAP kinase kinase 1 (PD 0325901, Axon Medchem, Groningen, the Netherlands) inhibitors (2i). The embryos were transferred to the 2i medium (N2B27 + ESGRO + 2i) until they developed into the blastocyst stage. The trophectoderm of the blastocyst was removed through immunosurgery (Solter & Knowles, 1975), and the inner cell mass was transferred first to the feeders in 2i medium and then after the Accutase treatment (Accutase: Gibco™ StemPro™ Accutase™ Fisher Scientific) on gelatinized (Gelatin, Sigma‐Aldrich, St. Louis, MO) plates in 2i medium supplemented with 100 μ/ml penicillin–streptomycin (Sigma‐Aldrich, St. Louis, MO).

The ES cells were transferred to gelatinized (Millipore, Billerica, MA) plates, and the medium was changed to a complete basal medium (Millipore, Billerica, MA) supplemented with a 2i Supplement Kit (Millipore, Billerica, MA) and 100 μ/ml penicillin–streptomycin (Sigma‐Aldrich, St. Louis, MO). The medium was changed daily, and Accutase (Millipore, Billerica, MA) was used for passaging the cells. After passage four, the cells did not attach to the gelatinized plate and were cultured in suspension thereafter. The cells were harvested for RNA and protein samples from a 10‐cm cell culture dish at passages four and five, respectively.

### 4.5 Immunoblotting

The protein isolation and immunoblotting protocol used has been described in detail previously (Hiltunen et al., 2020). In brief, ES cell pellets were solubilized with 1.5% dodecyl β‐d‐maltopyranoside (Sigma‐Aldrich, St. Louis, MO) with a protease inhibitor cocktail (Thermo Fisher Scientific, Rockford, IL). The amount of protein in the cell and tissue lysates was measured using a Coomassie protein assay (Thermo Fisher Scientific, Rockford, IL) and a FLUOstar Omega microplate reader (BMG LabTech, Ortenberg, Germany). A total of 20 μg of protein was run on 4%–20% polyacrylamide gel (Bio‐Rad, Hercules, CA) in a tris/glycine/SDS buffer (BioRad, Hercules, CA). The proteins were transferred onto a nitrocellulose membrane (Bio‐Rad, Hercules, CA) using a Trans‐blot Turbo Transfer System (Bio‐Rad, Singapore). The membrane was blocked with 5% non‐fat dry milk (Valio, Finland) in Tris‐buffered saline (Medicago, Uppsala, Sweden) with 0.01% Tween 20 (Fisher Scientific, Geel, Belgium) and an ECL kit (Advansta, Menlo Park, CA). A LAS‐3000 Luminescent Image Analyzer (Fuji Photo Film, Tokyo, Japan) was used to detect bands after antibody incubations. Fiji software was used to determine the band intensities (Schindelin et al., 2012).

The primary antibodies used were NHLRC2 antibody (Novus Biologicals, NBP1‐85019, 0.2 μg/ml) and glyceraldehyde‐3‐phosphate dehydrogenase (GAPDH) antibody (GeneTex, GTX100118, 0.1 μg/ml). Goat Anti‐Rabbit IgG H&L horseradish peroxidase (Abcam ab97080, 0.05 μg/ml) was used as a secondary antibody.

### 4.6 Quantitative PCR

RNA was isolated using an RNase plus mini kit (Qiagen, Hilden, Germany) and RNase‐free DNase (Qiagen, Hilden, Germany) according to the manufacturer's instructions.

A QuantiTect Reverse Transcription Kit (Qiagen, Hilden, Germany) set was used for cDNA synthesis according to the manufacturer's instructions. qPCR primers (Table 3) were designed using NCBI Primer‐BLAST (Ye et al., 2012), and the Tm used was 60°C. qPCR was performed according to the manufacturer's instructions (IQTM SYBR Green Supermix, Bio‐Rad, Hercules, CA) with a CFX ConnectTM Real‐Time System (Bio‐Rad, Hercules, CA). *Gapdh* and *Actb* were used as reference genes in undifferentiated cells and *Gapdh* and *Pkg1* in spontaneously differentiated ES cells (Willems et al., 2006).

**TABLE 3:** qPCR primers

Gene	Sequence (5′–3′)
Oct4_F	CAACTCCCGAGGAGTCCCA
Oct4_R	CTGGGTGTACCCCAAGGTGA
Nanog_F	CAGAAAAACCAGTGGTTGAAGA
Nanog_R	GCAATGGATGCTGGGATACTC
Sox2_F	CACAGATGCAACCGATGCA
Sox2_R	GGTGCCCTGCTGCGAGTA
Actb_F	GCTGTATTCCCCTCCATCGTG
Actb_R	CACGGTTGGCCTTAGGGTTCAG
Gapdh_F	CCCCAATGTGTCCGTCGTG
Gapdh_R	GCCTGCTTCACCACCTTCT
Nestin_F	CTCTTCCCCCTTGCCTAATACC
Nestin_R	TTTAGGATAGGGAGCCTCAGACAT
Gata6_F	GAAGCGCGTGCCTTCATC
Gata6_R	GTAGTGGTTGTGGTGTGACAGTTG
Actc1_F	CCAAAGCTGTGCCAGGATGT
Actc1_R	GCCATTGTCACACACCAAAGC
Pkg1_F	CTGACTTTGGACAAGCTGGACG
Pgk1_R	GCAGCCTTGATCCTTTGGTTG

### 4.7 Isolation of E6.5–E8.5 embryos

Embryos were dissected in Dulbecco's Modified Eagle Medium (DMEM) (Corning, Mediatech, Manassas, VA) supplemented with 10% fetal bovine serum (FBS) (Pan‐Biotech, Aidenbach, Germany) and 25 mM HEPES (Gibco, Life Technologies Limited, Paisley, UK), as described previously (Nagy, Gertsenstein, Vintersten, & Behringer, 2003).

### 4.8 Light microscopy

The 1% low‐melt agarose (ROTH, Karlsruhe, Germany)‐embedded embryos were imaged using a Leica MZ6 stereo microscope equipped with a Leica DFC425 camera (Leica Microsystems GmbH, Wezlar, Germany) or a Zeiss Axio Zoom V16 macroscope equipped with an Axiocam 305 color camera, as well as a PlanNeoFluar Z 2.3×/0.57 objective (Carl Zeiss GmbH, Oberkochen, Germany). Transmitted light bright‐field and oblique illumination were used as contrast methods. Embryo clearing for β‐galactosidase‐stained samples was performed using 1:2 benzoic alcohol–benzyl benzoate (BABB) (Merck, Darmstadt, Germany) for 3 hr after dehydration through an alcohol series (50% ethanol, 95% ethanol, absolute ethanol, and methanol).

### 4.9 Histology

Decidua were dissected on E7.5‐8.5 and processed as described previously (Hiltunen et al., 2020). The decidua were fixed with 10% neutral buffered formalin (FF Chemicals, Haukipudas, Finland) for 24 hr at room temperature under agitation. Tissues were processed using Tissue‐Tek VIP 5 Jr, embedded in paraffin, and sectioned into 5‐μm sections (Microm, Walldorf, Germany). The sections were stained with hematoxylin and eosin and imaged with a NanoZoom S60 scanner (Hamamatsu, Hamamatsu City, Japan) at ×20 or ×40 magnification.

### 4.10 Transmission electron microscopy

The embryos were fixed with 1% glutaraldehyde and 4% formaldehyde in 0.1 M phosphate buffer (pH 7.4) for 20 min and embedded in low‐melt agarose. Agarose‐embedded embryos were then prepared and imaged at the Biocenter Electron Microscopy Core Facility (Oulu, Finland). The embryos were postfixed with 1% osmium tetroxide (Electron Microscopy Sciences, Hatfield, PA), dehydrated in acetone, and embedded in Epon LX112 (#21210; Ladd Research Industries Inc., Williston, VT). Hydroxypropyl methacrylate (Sigma, St. Louis, MO) was used in the embedding of β‐galactosidase‐stained embryos to stabilize the X‐gal reaction product (Masahira et al., 2005). Thin and semi‐thin sections were cut throughout the embryo using a Leica Ultracut UCT microtome (Leica, Wetzlar, Germany), and toluidine blue‐stained semi‐thin sections were used to select thin sections. The thin sections were stained with uranyl acetate and lead citrate and examined using a Tecnai G2 Spirit 120 kV transmission electron microscope (FEI, Eindhoven, Netherlands). Images were captured using a Quemesa CCD camera (Olympus Soft Imaging Solutions GMBH, Münster, Germany).

### 4.11 Spontaneous differentiation of mouse ES cells

A sample of 5 × 10^6^ ES cells were plated into two wells of an uncoated six‐well plate at passage five. The medium was changed at Day 2. On Day 3, the formed embryoid bodies were transferred to a 6‐cm plate coated with gelatin, and the medium was changed to a differentiation medium: complete basal medium (Millipore, Billerica, MA) with 10% FBS (Pan‐Biotech, Aidenbach, Germany) and 100 μ/ml penicillin–streptomycin (Sigma‐Aldrich, St. Louis, MO). The medium was changed every other day, and the cells were collected for RNA isolation after 16 days.

### 4.12 β‐Galactosidase staining

The embryos were stained using a β‐galactosidase staining kit (Biovision, Milpitas, CA) according to the manufacturer's instruction. The stained embryos were postfixed for 10 min with 4% paraformaldehyde and mounted in 1% low‐melt agarose (ROTH, Karlsruhe, Germany). For histological sectioning, the embryos were embedded in paraffin after dehydration in ethanol and isopropanol and sectioned into 6‐μm sections using a microtome (Microm, Walldorf, Germany). The embryos were counterstained in eosin for 1.5 min with a minimal xylene treatment to avoid the loss of the X‐gal staining product.

## Data Availability

The data generated or analyzed during this study are included in this published article. Further information and additional datasets are available from the corresponding author upon reasonable request.
